# Tamalin Function Is Required for the Survival of Neurons and Oligodendrocytes in the CNS

**DOI:** 10.3390/ijms232113395

**Published:** 2022-11-02

**Authors:** Yongbo Seo, Seojung Mo, Suhyun Kim, Hyun Kim, Hae-Chul Park

**Affiliations:** 1 Department of Biomedical Sciences, Korea University College of Medicine, Seoul 02841, Korea; 2 Department of Anatomy, Korea University College of Medicine, Seoul 02841, Korea

**Keywords:** tamalin, arf6, neurodegeneration, oligodendrocytes, mGluR5, zebrafish, mice

## Abstract

Tamalin is a post-synaptic scaffolding protein that interacts with group 1 metabotropic glutamate receptors (mGluRs) and several other proteins involved in protein trafficking and cytoskeletal events, including neuronal growth and actin reorganization. It plays an important role in synaptic plasticity in vitro by controlling the ligand-dependent trafficking of group 1 mGluRs. Abnormal regulation of mGluRs in the central nervous system (CNS) is associated with glutamate-mediated neurodegenerative disorders. However, the pathological consequences of tamalin deficiency in the CNS are unclear. In this study, tamalin knockout (KO) zebrafish and mice exhibited neurodegeneration along with oligodendrocyte degeneration in the post-embryonic CNS to adulthood without any developmental defects, thus suggesting the function of tamalin is more important in the postnatal stage to adulthood than that in CNS development. Interestingly, hypomyelination was independent of axonal defects in the CNS of tamalin knockout zebrafish and mice. In addition, the loss of Arf6, a downstream signal of tamalin scaffolding protein, synergistically induced neurodegeneration in tamalin KO zebrafish even in the developing CNS. Furthermore, tamalin KO zebrafish displayed increased mGluR5 expression. Taken together, tamalin played an important role in neuronal and oligodendrocyte survival and myelination through the regulation of mGluR5 in the CNS.

## 1. Introduction

Tamalin is a scaffold protein consisting of multiple protein–protein interaction domains. It is composed of a post synaptic density protein (PSD95), Drosophila disc large tumor suppressor (Dlg1), and zonula occludens-1 protein (zo-1) (PDZ) domain, a proline-rich region, a leucine zipper region, and a C-terminal PDZ-binding motif. The PDZ domain interacts with the C terminus of group I metabotropic glutamate receptors (mGluRs) [1] and a truncated isoform of the neurotrophin-3 receptor, tropomyosin receptor kinase C [2]. In addition, tamalin interacts with other important scaffold proteins involved in post-synaptic organization and protein trafficking in neurons [3,4]. In immature hippocampal neurons, tamalin knockdown markedly reduces dendritic outgrowth, the number of dendritic tips, and the levels of filamentous actin and microtubule-associated protein 2 in dendrites, thus suggesting its role in neuronal dendritic development via the regulation of EFA6A/ADP-ribosylation factor 6 (Arf6)-mediated cytoskeleton dynamics [5]. Interestingly, tamalin knockout (KO) mouse do not exhibit any developmental defects and develop normally [6,7]. However, tamalin deficiency significantly reduces the sensitivity to acute morphine responses, impairs adaptive responses to morphine and cocaine [6], and abrogates electroconvulsive shock (ECS)-induced neurogenesis in the adult mouse hippocampus [7]. The multiple protein domains of tamalin interact with several neuronal proteins involved in synaptic transmission and neuronal development; nonetheless, the absence of neurodegenerative defects in the central nervous system (CNS) because of tamalin dysfunction is unexpected. However, the pathological consequences of tamalin deficiency in the CNS are unclear.

Glutamate receptors are important for post-synaptic excitatory action that mediates glutamate in the nerve cells. mGluRs transmit signals through the G protein and can be largely classified into groups 1, 2, and 3. Group 1 mGluRs, which include mGluR1 and mGluR5, mediate slow excitatory neurotransmission in the CNS and play critical roles in various neuronal functions. The abnormal regulation of glutamate associated with group 1 mGluRs leads to the excitatory toxicity of neuronal cells induced by calcium influx, which is associated with several neurological diseases, such as Alzheimer’s disease, Parkinson’s disease, and amyotrophic lateral sclerosis (ALS) [8,9]. In addition, the excitotoxicity of glutamate can cause demyelination and cell death in diseases, such as multiple sclerosis (MS) [10,11]. mGluR1 is strongly expressed in demyelinated axons in patients with MS, thus suggesting that abnormal expression of mGluR in axons affects the pathology of myelination [12]. Recently, an in vitro study demonstrated that tamalin plays a critical role in the ligand-dependent internalization of mGluR1 and mRgluR5, and the knockdown of endogenous tamalin inhibits the ligand-dependent internalization of these receptors [13]. Thus, altered tamalin function may be involved in neurodegeneration through the dysregulation of mGluRs.

In this study, we aimed to investigate tamalin KO zebrafish and mice to determine its role in the CNS. By investigating its function in developing and postnatal CNS, this novel study aimed to provide compelling evidence for the crucial role of tamalin in neuronal and oligodendrocyte survival and myelination through the regulation of mGluR5 in the embryonic and post-embryonic CNS.

## 2. Results

### 2.1. Tamalin Is Required for the Survival of Neurons in the Postembryonic CNS

**Figure 1:**
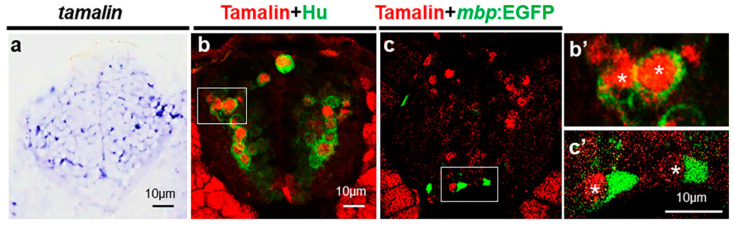
Tamalin is expressed in the neurons but not in the oligodendrocytes. (**a**–**c’**) The representative images are transverse sections of the spinal cord. The dorsal side is displayed at the top. (**a**) Whole-mount in situ RNA hybridization with tamalin mRNA in the spinal cord of wildtype zebrafish at 3 dpf. (**b**,**b’**) Immunolabeling of wildtype zebrafish with anti-Hu and anti-tamalin antibodies, and (**c**,**c’**) the immunolabeling of *Tg(mbp:egfp)* zebrafish with an anti-Tamalin antibody at 5 dpf. (**b’**,**c’**) High magnification images of the boxes in (**b**,**c**). Asterisks indicate the tamalin^+^ cells. Scale bars: (**a**–**c’**), 10 μm. EGFP, Enhanced green fluorescent protein; dpf, days post fertilization.

To investigate the function of tamalin, we first examined its expression in zebrafish. Whole-mount in situ RNA hybridization revealed that tamalin was generally expressed in the spinal cord (Figure 1a). Immunohistochemistry with anti-tamalin and anti-Hu antibodies, which identify tamalin and postmitotic neurons, respectively, demonstrated the expression of tamalin in neurons (Figure 1b,b’). We did not detect tamalin expression in mature oligodendrocytes, which were marked by myelin basic protein (*mbp*):EGFP expression in the spinal cord of *Tg(mbp:egfp)* embryos [14] (Figure 1c,c’), thus indicating that in the postnatal CNS, tamalin is expressed in neurons but not in oligodendrocytes. 

To investigate the loss of tamalin function, we generated tamalin KO zebrafish using the Crispr/Cas9 system (Figure S1). Vertebrate tamalin consists of a PDZ domain, leucin zipper, and proline rich domain (Figure S1a). Of these domains, the PDZ domain interacts with the group 1 mGluRs and binds to postsynaptic density protein 95 and synaptic scaffolding molecule to form a scaffolding protein complex [4]. The amino acid sequence of the zebrafish PDZ domain is conserved in humans and mice with 70% sequence similarity (Figure S1b). We designed a guide RNA to target the PDZ domain and performed genome editing with the Crispr/Cas9 system for a 12 bp insertion, which generated a premature stop codon and truncated the tamalin protein (Figure S1c). Moreover, we used a morpholino oligonucleotide against tamalin (tamalin MO) to knock-down its expression. Embryos injected with tamalin MO were not immunostained with the tamalin antibody, whereas the control embryos were stained, thereby indicating that tamalin MO specifically inhibited tamalin expression (Figure S1d,e).

**Figure 2:**
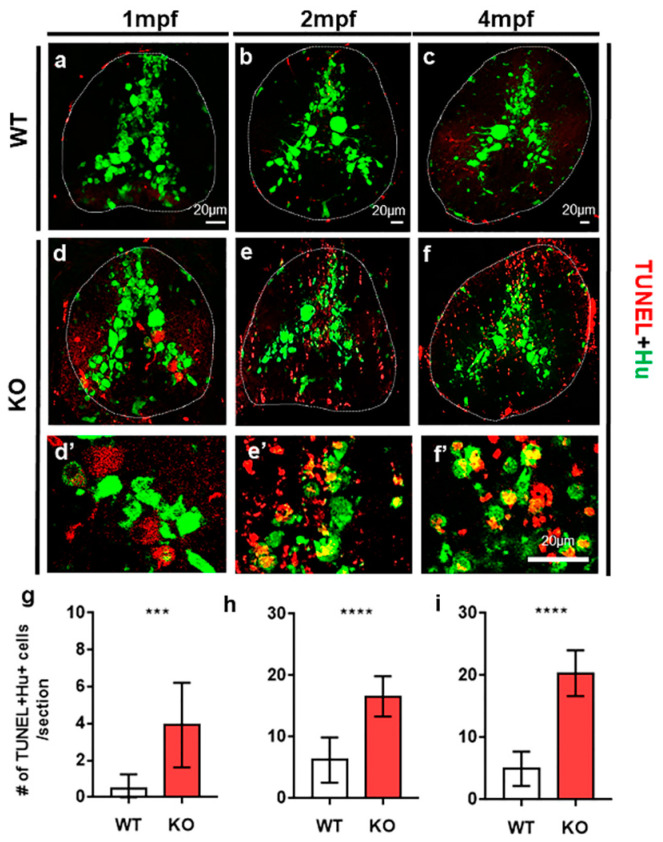
The loss of tamalin function causes neuronal cell death in the spinal cord of postembryonic zebrafish up to adulthood. (**a**–**f**) The representative images are transverse sections of the spinal cord of wildtype (**a**–**c**) and tamalin KO zebrafish (**d**–**f**) labeled with anti-Hu antibody (green) and TUNEL staining (red). (**d’**–**f’**) High magnification images of the boxed area in (**d**–**f**) show TUNEL+ Hu+ dying neurons. The dorsal side is displayed at the top. (**g**–**i**) Quantification of the number of TUNEL+ Hu+ cells in the wildtype or tamalin KO zebrafish at 1 mpf, 2 mpf, and 4 mpf (*** *p* = 0.0007 **** *p* < 0.0001, *n* = 10 sections from five zebrafish). Scale bars: (**a**–**f**), 20 µm. KO, knockout; mpf; months post fertilization; and TUNEL, terminal deoxynucleotidyl transferase dUTP nick end labeling.

Subsequently, we examined neuronal and oligodendrocyte development in the absence of tamalin function. Immunolabeling neurons with anti-Hu antibody (Figure S2a–d), the fluorescent labeling of oligodendrocytes with *mbp*:EGFP expression in *Tg(mbp:egfp)* (Figure S2e–h), and the fluorescent labeling of myelin sheath with *claudinK*:mGFP in *Tg(claudinK:mGFP)* embryos (Figure S2i–k) revealed normal neuronal and oligodendrocyte development in the spinal cord of tamalin KO- and tamalin MO-injected embryos. However, TUNEL staining for the detection of apoptotic cell death and anti-Hu antibody labeling displayed increased neuronal cell death in the spinal cord of 1-month-old post-embryonic tamalin KO zebrafish (Figure 2a,d,g). Neuronal apoptotic cell death continued to increase from 2 months post fertilization (mpf) (Figure 2b,e,h) to the adult stage at 4 mpf (Figure 2c,f,i). Thus, tamalin function was continuously required for the survival of neurons from the post-embryonic CNS to adulthood.

### 2.2. The Loss of Tamalin Function Caused Oligodendrocyte Cell Death and Hypomyelination

**Figure 3:**
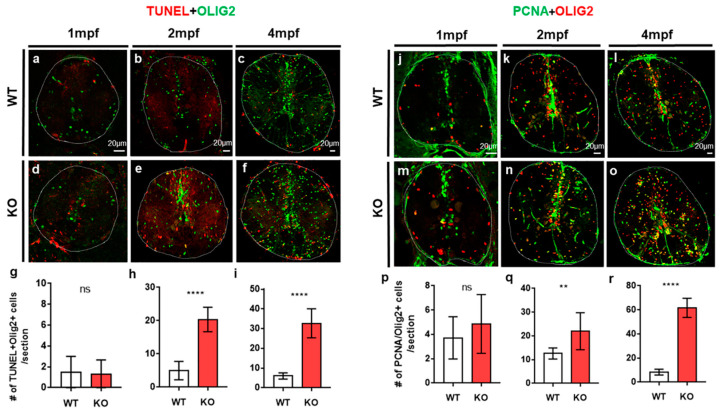
The loss of tamalin function causes cell death of oligodendrocyte lineage cells in the postembryonic zebrafish spinal cord to adulthood. The representative images are transverse sections of the spinal cord of post-embryonic zebrafish. The dorsal side is displayed at the top. (**a**–**f**) Immunolabeling of wildtype (**a**–**c**) and tamalin KO (**d**–**f**) zebrafish with anti-Olig2 antibody (green) and TUNEL staining (red). (**j**–**o**) Immunolabeling of wildtype (**j**–**l**) and tamalin KO zebrafish (**m**–**o**) with anti-PCNA (green) and anti-Olig2 (red) antibodies. Quantification of the number of TUNEL^+^ Olig2^+^ cells (**g**–**i**) and PCNA^+^ Olig2^+^ cells (**p**–**r**) at 1 mpf (**g**,**p**), 2 mpf (**h**,**q**), and 4 mpf (**i**,**r**). (** *p* = 0.002 **** *p* < 0.0001, *n* = 10 sections from five zebrafish). Scale bars: (**a**–**l**), 20 μm. KO, knockout; TUNEL, terminal deoxynucleotidyl transferase dUTP nick end labeling; PCNA, proliferating cell nuclear antigen; ns; no significance and mpf; months post fertilization.

TUNEL staining also revealed intensive apoptotic cell death of neurons as well as non-neuronal cells in the spinal cord of tamalin-KO zebrafish (Figure 2). To identify the cell types of non-neuronal cells that underwent apoptotic cell death, we performed TUNEL staining and immunostaining with anti-Olig2 antibody to identify the oligodendrocyte lineage cells, including oligodendrocyte progenitor cells (OPCs) and mature oligodendrocytes [15]. At 1 mpf, apoptotic cell death of Olig2^+^ oligodendrocyte lineage cells did not increase in the spinal cord of tamalin KO zebrafish, compared with controls (Figure 3a,d,g), whereas neuronal cell death increased at the similar stage (Figure 2a,d,g).

However, we observed substantially increased apoptotic cell death of Olig2^+^ cells in tamalin KO zebrafish (Figure 3b,e,h), as compared with the wildtype group. In addition, higher apoptotic cell death was observed in 4-month-old adult spinal cord of tamalin KO zebrafish as compared to that in the 2-month-old counterparts (Figure 3c,f,i). In other words, neuronal cell death occurred first, followed by oligodendrocyte cell death in the tamalin KO zebrafish. Immunostaining with anti-PCNA antibody, which detects proliferating cells, revealed similar numbers of PCNA^+^/Olig2^+^ proliferating OPCs in the spinal cord of wildtype and tamalin KO zebrafish at 1 mpf (Figure 3j,m,p). However, we observed an increased number of PCNA^+^/olig2^+^ proliferating OPCs in the spinal cord of the tamalin KO zebrafish at 2 mpf (Figure 3k,n,q), which was lower than that observed at 4 mpf (Figure 3l,o,r). To determine the impact of oligodendrocyte cell death on the reduction in mature oligodendrocytes, we investigated the number of *mbp*:EGFP^+^ mature oligodendrocytes in the *Tg(mbp:EGFP)*/tamalin^-/-^ zebrafish. The number of mature oligodendrocytes decreased at all stages examined in the spinal cord of tamalin KO zebrafish (Figure S3). Tamalin function was also required for the survival of oligodendrocytes. Further, oligodendrocyte cell death caused by loss of function may induce abnormal proliferation of OPCs to compensate for the loss of oligodendrocytes.

**Figure 4:**
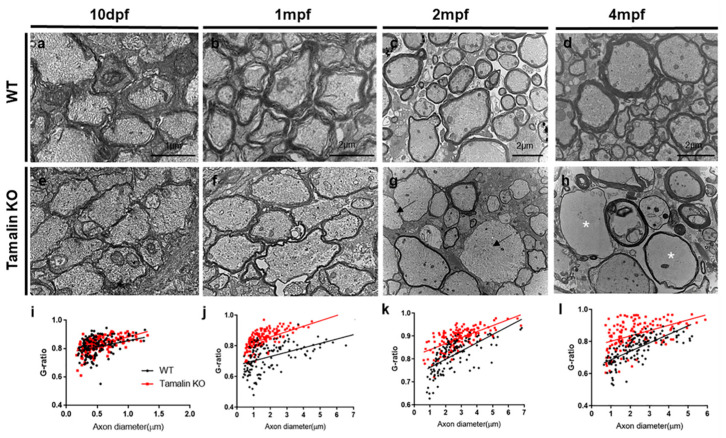
Tamalin KO causes the hypomyelination of axons in the zebrafish spinal cord. Transmission electron microscopy (TEM) images reveal transverse sections through the spinal cord of wildtype or tamalin KO zebrafish at 10 dpf (**a**,**e**), 1 mpf (**b**,**f**), 2 mpf (**c**,**g**), and 4 mpf (**d**,**h**). Black arrows indicate unmyelinated axons (**g**), and asterisks indicate disorganized watery axons (**h**). (**i**–**l**) The g-ratio of myelinated axons in the spinal cord of wildtype and tamalin KO zebrafish. Unpaired *t* test was used to compare means from each animal. Each g-ratio has been obtained from 100 myelinated axons in eight sections of four zebrafish each (**i**: *p* = 0.0696, **j**–**l**: *p* < 0.0001). Scale bars: (**a**,**e**): 1 μm, (**b**–**h**): 2 μm. KO, knockout; dpf, days post fertilization; and mpf; months post fertilization.

To better understand CNS defects caused by loss of tamalin function, we examined nerve ultrastructures in the spinal cords of tamalin KO zebrafish using TEM. Interestingly, examination of transverse spinal cord sections revealed that axons in tamalin KO zebrafish had similar thickness to that of a myelin sheath at 10 days post fertilization (dpf) (Figure 4a,e,i), but had thinner myelin sheaths and higher g-ratios than axons in control zebrafish at 1 mpf (Figure 4b,f,j). At 2 mpf, the myelin thickness was thinner than control as at 1 mpf in tamalin KO spinal cord, and some axons were non-myelinated (Figure 4c,g,k).

At 4 mpf, the tamalin KO spinal cord revealed numerous degenerations of the nerve ultrastructure due to apoptotic cell death of neurons and oligodendrocytes (Figure 4d,h,i). Notably, maximum axons displayed watery degeneration, and the subcellular organelles were completely lost (Figure 4h, white asterisks). Taken together, these results show that tamalin function is required for the survival of neurons and oligodendrocytes in the postembryonic CNS of zebrafish. In addition, observation of the hypomyelinated or non-myelinated axons without pathological hallmarks of neurodegeneration in the spinal cord of tamalin KO suggest that, although tamalin is expressed in neurons but not in oligodendrocytes, tamalin function is also involved in oligodendrocyte myelination.

**Figure 5:**
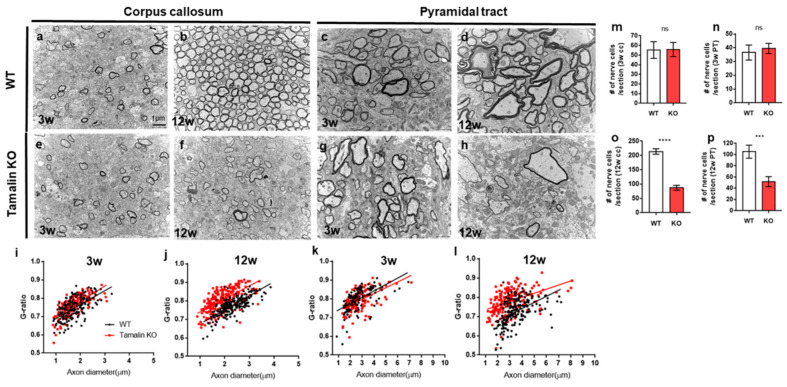
Tamalin KO induces nerve degeneration and hypomyelination in the postembryonic mouse brain. (**a**–**h**) TEM images displaying transverse sections of the corpus callosum (**a**,**b**,**e**,**f**) and pyramidal tract (**c**,**d**,**g**,**h**) at 3 weeks (**a**,**e**,**c**,**g**) and 12 weeks (**b**,**f**,**d**,**h**) in wildtype and tamalin KO mice. (**i**–**l**) The g-ratio of myelinated axons in the corpus callosum (**i**,**j**) and pyramidal tract (**k**,**l**) of wildtype and tamalin KO zebrafish. Unpaired *t* test was used to compare means from each animal. Each g-ratio was from 100 myelinated axons in eight sections of four mice each (**i**: *p* = 0.0535, **k**: *p* = 0.0579, **j**,**l**: **** *p* < 0.0001). (**m**–**p**) Quantification of the number of nerve cells in the corpus callosum (**m**,**o**) and pyramidal tract (**n**,**p**) of wildtype and tamalin KO mice (**** *p* < 0.0001, *** *p* < 0.001). ns; no significance. Scale bars: (**a**–**h**): 1 μm. KO, knockout; TEM, transmission electron microscopy.

To test whether tamalin function is similar between zebrafish and mammals, we subsequently investigated its loss of function by analyzing the corpus callosum (CC) and pyramidal tract (PT) in the cerebral cortex of tamalin KO mice [7] (Figure 5). Three-week-old controls and tamalin KO mice had similar numbers of nerve fibers (Figure 5a,e,m,c,g,n), which considerably decreased in 12-week-old tamalin KO mice as compared to the wildtype (Figure 5b,f,o,d,h,p). Therefore, there was no apoptotic cell death of neurons and oligodendrocytes in the CNS of 3-week-old tamalin-KO mice; however, cell death occurred after 3 weeks of age and the number of nerve fibers decreased at 12 weeks.

Moreover, we analyzed CC and PT myelination in Tamalin KO mice, and the majority of the axons were normally myelinated in CC and PT at 3 weeks of age (Figure 5a,e,i,c,g,k). However, maximum axons in tamalin KO mice were unmyelinated or hypomyelinated with increased g-ratio, as compared with controls at 12 weeks of age (Figure 5b,f,j,d,h,l). Taken together, tamalin function was required for maintaining myelination, in addition to neuronal and oligodendrocyte survival in the post-embryonic CNS of zebrafish and mice. To examine the role of tamalin in behavior, we performed the elevated plus maze (EPM) test. Mice with the *tamalin* gene knockout (KO) showed decreased number of open arm entries and time spent in open arms compared with the wild-type mice in the EPM. Thus, these results suggest that tamalin deletion leads to anxiety-like behavior in mice (Figure S4).

### 2.3. The Loss of Tamalin and Arf6 Synergistically Induced Neurodegeneration through Glutamate Toxicity

Arf6 is a GTP binding protein and works as a downstream signal of the tamalin scaffolding protein, thus activating actin reorganization or membrane trafficking in the cell membrane via Tamalin-Arf6-Rac1 signaling [2]. To determine if the loss of Arf6 function synergistically induced neurodegeneration in the spinal cord of tamalin KO zebrafish, we first examined the expression of Arf6 in zebrafish. Fluorescent in situ RNA hybridization with Arf6 and co-labeling with anti-Hu antibody immunostaining in *Tg(mbp:egfp)* zebrafish revealed that Arf6 was exclusively expressed in the neurons but not in the oligodendrocytes, similar to tamalin (Figure S5a,b’).

**Figure 6:**
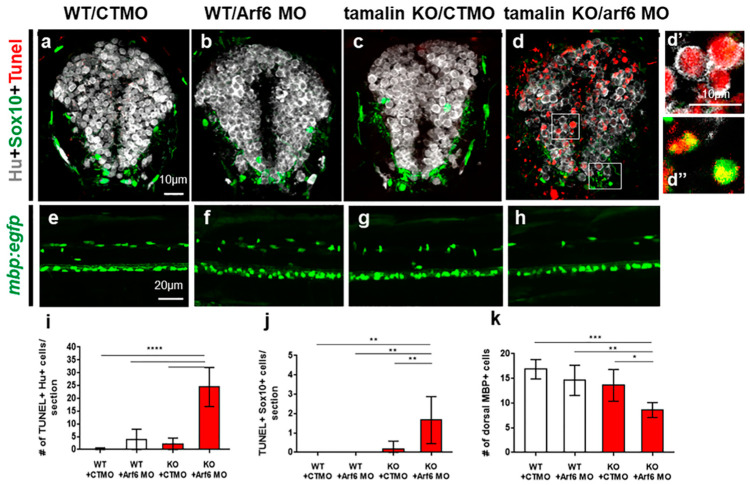
The loss of tamalin and Arf6 synergistically induces neuronal and oligodendrocyte degeneration during CNS development. (**a**–**d**) The representative images are transverse sections of the spinal cord at 3 dpf. The dorsal side is displayed at the top. Wildtype embryos injected with control MO (CTMO) (**a**) and Arf6 MO (**b**), or tamalin KO embryos injected with CTMO (**c**) and Arf6 MO (**d**) have been immunolabeled with anti-Hu (white) and anti-Sox10 (green) antibodies to detect neurons and oligodendrocyte lineage cells, respectively, and TUNEL staining (red) to detect apoptotic cell death. (**d’**,**d”**) High magnification images of the boxes in (**d**). (**e**–**h**) Lateral views of the spinal cord of 3 dpf *Tg(mbp:egfp)* zebrafish injected with CTMO (**e**) and Arf6 MO (**f**), or tamalin KO zebrafish injected with CTMO (**g**) and Arf6 MO (**h**). The anterior side is displayed on the left. (**i**–**k**) Quantification of the number of TUNEL+/Hu+ neurons (**i**), TUNEL+/Sox10+ oligodendrocyte lineage cells (**j**) and MBP+ mature oligodendrocytes (**k**). (**** *p* < 0.0001, *** *p* < 0.001, ** *p* < 0.01, * *p <* 0.05). Scale bars: (**a**–**d”**), 10 μm (**e**–**h**), 20 μm (**a**–**d**: *n* = 10 sections from five zebrafish, (**e**–**h**): each image from ten zebrafish). KO, knockout; MO, morpholino oligonucleotides; CNS, central nervous system; dpf, days post fertilization; TUNEL, terminal deoxynucleotidyl transferase dUTP nick end labeling; Sox10, Sry-related HMg-Box gene 10; Arf6, ADP-ribosylation factor 6; and MBP, myelin basic protein.

To investigate the loss of Arf6 function, we used morpholino oligonucleotides (Arf6 MO) to simultaneously knock-down both Arf6a and Arf6b expression by blocking translation. To assess the specificity of Arf6 MO, we generated *hsp70:arf6a-mcherry* and *hsp70:arf6b-mcherry* DNA constructs, which expressed Arf6a/6b-mCherry fusion protein under the control of heat-shock inducible promoter (hsp70). Consequently, *hsp70:arf6a/6b-mCherry* DNAs were injected into the one-cell-stage zebrafish embryos with either control MO or Arf6 MO, and the injected embryos were exposed to a heat shock to induce the expression of exogenous Arf6-mCherry fusion protein. Arf6 MO significantly reduced the intensity of mCherry fluorescence, thus indicating the effective reduction in Arf6a/6b protein expression (Figure S5c–f). Subsequently, we determined the synergistic effects of tamalin and Arf6 in neurodegeneration (Figure 6).

The spinal cord of wildtype embryos injected with CTMO (Figure 6a,e), Arf6 MO (Figure 6b,f), and tamalin KO embryos injected with CTMO (Figure 6c,g) did not show TUNEL^+^ dying cells and had a normal number of mbp:EGFP^+^ oligodendrocytes, which indicated that the loss of tamalin or ARF6 alone did not induce neurodegeneration in the developing CNS. However, tamalin KO embryos injected with Arf6 MO revealed intensive TUNEL^+^ apoptotic cell death of Hu^+^ neurons (Figure 6d,d’,i) and Sox10^+^ oligodendrocytes (Figure 6d,d”,j), thereby reducing mature oligodendrocytes in the spinal cord of *Tg(mbp:egfp)/tamalin^-/-^* zebrafish (Figure 6h,k). The loss of Arf6 function synergistically induced neurodegeneration in the spinal cord of tamalin KO zebrafish.

**Figure 7:**
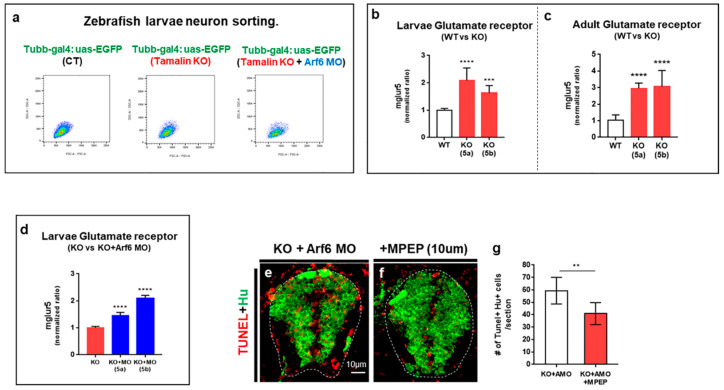
The loss of tamalin and Arf6 synergistically induces the upregulation of glutamate receptor expression. (**a**) The isolation of tubb:EGFP+ neurons from the spinal cord of *Tg(tubb:gal4;uas:egfp)*, *Tg(tubb:gal4;uas:egfp)*/*tamalin*^-/-^, and *Tg(tubb:gal4;uas:egfp)*/*tamalin*^-/-^/*arf6* MO-injected embryos by fluorescent activated cell sorting (FACS). (**b**) qRT-PCR analysis to compare *mglur5a/5b* expression level in the neurons isolated from the spinal cord of *Tg(tubb:gal4;uas:egfp)* and *Tg(tubb:gal4;uas:egfp)*/*tamalin*^-/-^ embryos. (**c**) qRT-PCR analysis to compare *mglur5a/5b* expression levels in the spinal cord of adult *Tg(tubb:gal4;uas:egfp)* and *Tg(tubb:gal4;uas:egfp)*/*tamalin*^-/-^ zebrafish (*** *p* < 0.001 **** *p* < 0.0001). (**d**) qRT-PCR analysis to compare neuronal *mglur5a/5b* expression level isolated from the spinal cord of *Tg(tubb:gal4;uas:egfp)*/*tamalin*^-/-^ and *Tg(tubb:gal4;uas:egfp)*/*tamalin*^-/-^/*arf6* MO-injected larvae (**** *p* < 0.0001). (**e**,**f**) Transverse sections of the spinal cord of tamalin KO/Arf6 MO-injected embryos not treated € and treated with MPEP (**f**). Immunolabeling with anti-Hu antibody (green) and TUNEL staining (red) has been performed to detect neuronal apoptotic cell death. (**g**) Quantification of the number of Tunel^+^ Hu^+^ cells. (** *p* = 0.01). (**e**,**f**): n = 10 sections from five zebrafish. Scale bars: (**e**,**f**), 10 μm. EGFP, enhanced green fluorescent protein; MO, morpholino oligonucleotides; Qrt-PCR, quantitative real time polymerase chain reaction; Arf6, ADP-ribosylation factor 6; TUNEL, terminal deoxynucleotidyl transferase dUTP nick end labeling; and MPEP, 2-Methyl-6-(phenylethynyl)-pyridine.

A recent in vitro study has demonstrated the importance of tamalin for the endocytosis and trafficking of mGluR1 [13]; therefore, we hypothesized that the loss of tamalin function induced neurodegeneration through glutamate excitotoxicity, which was caused by the abnormal regulation of glutamate and its receptors. First, we isolated neurons from *Tg(tubb:gal4::uas:egfp)*, *Tg(tubb:gal4::uas:egfp)*/*tamalin^-/-^*, and *Tg(tubb:gal4::uas:egfp)/tamalin*^-/-^/*arf6*-MO injected embryos, which expressed enhanced green fluorescent protein (EGFP) in the neurons, as detected by fluorescent activated cell sorting (FACS) (Figure 7a). Subsequently, we analyzed the mRNA levels of *mglur5a/5b* by quantitative real-time PCR (qRT-PCR) in the isolated neurons. The mRNA levels of *mglur5a* and *mglur5b* were significantly increased in the neurons from tamalin KO as compared to wildtype (Figure 7b).

To compare the mRNA levels of *mglur5a/5b* in adults, we performed qRT-PCR analysis using the RNAs isolated from the adult spinal cord of wildtype and tamalin KO zebrafish. The mRNA levels of *mglur5a/5b* had increased significantly in the spinal cord of tamalin KO zebrafish (Figure 7c). Furthermore, mRNA levels of *mglur5a/5b* in the neurons of tamalin KO injected with Arf6 MO displayed greater increase than that of tamalin KO (Figure 7d). Because there is no antibody to detect zebrafish mGluR5 protein, we could not quantify the mGluR5 protein to confirm the change in mGluR5 in the absence of tamalin and Arf6. To determine if higher levels of glutamate receptors induced excitotoxicity in neurons due to the loss of tamalin and Arf6, we treated tamalin KO embryos injected with Arf6 MO with 10 mM of 2-Methyl-6-(phenylethynyl)-pyridine (MPEP), which is a highly selective antagonist of the mGlu5 receptor [16]. Conceivably, TUNEL+ apoptotic cell death decreased in the MPEP-treated tamalin KO embryos injected with Arf6 MO, thus indicating that glutamate excitotoxicity caused by increased mGluR5 levels induced early apoptotic cell death in the tamalin KO/arf6 MO embryos (Figure 7e–g). Taken together, the loss of tamalin and Arf6 function synergistically contributed to neurodegeneration through glutamate excitotoxicity.

## 3. Discussion

Tamalin KO mice develop normally and do not exhibit any defects in their physical characteristics and behaviors under normal conditions. Considering the interaction between multiple protein domains of tamalin and neuronal proteins that are involved in synaptic transmission and neuronal development [1,2,3,4,17], the absence of behavioral changes under normal conditions or neurodegenerative defects in the CNS because of tamalin dysfunction was unexpected. Consistent with a previous tamalin KO study, our findings demonstrated that tamalin KO zebrafish and mice did not display any developmental defects. However, they exhibited neurodegeneration and oligodendrocyte degeneration in the post-embryonic CNS to adulthood. The increase in the number of TUNEL+ neurons and oligodendrocytes in tamalin KO zebrafish suggests that tamalin deficiency can induce cell death through apoptosis, a tightly regulated form of programmed cell death. Therefore, these data suggest that tamalin function is more important from the postnatal stage to adulthood than that in CNS development. Our findings were supported by previous studies suggesting that tamalin mRNA expression levels in the mouse brain were low before and at birth, but continued to increase during the postnatal period [4]. Moreover, tamalin deletion in mice abrogated ECS-induced neurogenesis in the adult mouse hippocampus, without inducing any developmental defects. This suggested that tamalin played a greater role from postnatal stages to adulthood than that in CNS development [7]. Interestingly, the loss of Arf6, which acts as a downstream signal of tamalin scaffolding protein, synergistically induced neurodegeneration in tamalin KO even in the developing CNS, thus indicating the need for tamalin function for the survival of neurons and oligodendrocytes in the embryonic CNS, in addition to its role in the post-embryonic CNS.

Group I mGluRs, which include mGlu1 and mGlu5, mediate slow excitatory neurotransmission in the CNS and play critical roles in various neuronal functions. The abnormal regulation of group 1 mGluRs in the CNS has been implicated in glutamate-mediated neuropsychiatric conditions ranging from neurodevelopmental to neurodegenerative disorders, such as Alzheimer’s disease and ALS [18,19,20,21,22]. Particularly, the activation of mGluR1/5 by agonist treatment produces abnormal glutamate release, thus causing glutamate excitotoxicity in a mouse model of ALS [23], whereas the reduction in mGluR1 expression revealed prolonged survival probability, delayed pathology onset, and slower disease progression [24].

mGluRs are strongly regulated by a protein complex at the post-synaptic membrane, which comprises tamalin as a key component [13,25,26,27]. Taken together, altered tamalin function may be involved in neurodegeneration through dysregulation of mGluRs. This novel study provided compelling evidence that the loss of tamalin function induced neurodegeneration along with oligodendrocyte degeneration, besides increased mGluR5a/5b expression in the neurons of tamalin KO zebrafish. Furthermore, treatment with the mGluR5 antagonist MPEP reduced apoptotic cell death in Arf6 MO-injected tamalin KO zebrafish, thus suggesting the dysregulation of mGluR5 owing to tamalin loss induced neurodegeneration. Similarly, mGluR5 protein levels were significantly higher in the prefrontal cortex of patients with schizophrenia, whereas mGluR5 regulatory proteins, such as tamalin and norbin, were expressed at lower levels in these patients as compared to controls [28]. This study supported the idea that the dysregulation of mGluR5 mediated by altered tamalin expression contributed to neurological disorders.

Moreover, we demonstrated that the loss of Arf6 function synergistically induced neurodegeneration even in the developing spinal cord of tamalin KO zebrafish. In addition, the mRNA level of mglur5a/5b in arf6 MO-injected tamalin KO neurons displayed a greater increase than that in tamalin KO neurons, thus suggesting that tamalin was associated with Arf6 in controlling mGluR5 expression in neurons. Previously, we demonstrated that tamalin colocalizes with Arf6 and is responsible for neuronal dendritic development via the regulation of EFA6A/Arf6-mediated cytoskeleton dynamics [5]. Furthermore, cytohesin-2, a guanine nucleotide exchange factor for Arf6, is functionally associated with mGluR5 during the development of mechanical allodynia through the activation of Arf6 in the mouse spinal cord, thereby suggesting that Arf6 is also associated with the regulation of mGluR5 [29].

Tamalin is expressed only in neurons and not in oligodendrocytes; however, we observed hypomyelination of the healthy axons in tamalin KO zebrafish and mice. We hypothesized that tamalin could affect CNS myelination through Arf6, which forms a protein complex with tamalin. Our hypothesis was supported by previous studies suggesting that Arf6 is involved in myelination in the CNS and peripheral nervous system [30,31,32,33]. Particularly, the analysis of conditional knock-out (CKO) mice lacking Arf6 in the neurons revealed impaired axonal myelination in the brain of neuron-specific Arf6 CKO mice. In addition, neuronal Arf6 regulates OPC migration and differentiation by promoting neuronal secretion of fibroblast growth factor-2, a guidance factor for OPC migration [30]. Taken together, the neuronal expression of tamalin and Arf6 played an important role in oligodendrocyte myelination by indirectly regulating OPC migration and differentiation.

## 4. Materials and Methods

### 4.1. Ethics Statement

All experimental procedures were approved by the Korea University Institutional Animal Care and Use Committee, and were performed in accordance with the animal experiment guidelines of the Korea National Veterinary Research and Quarantine Service.

### 4.2. Fish Lines and Mouse Lines

Wild-type AB, *Tg(mbp:egfp)* [14], *Tg(cldnk:gal4vp16;uas:egfpCAAX*) [34], *Tg(Tubb:gal4vp16)* and *Tg(uas:egfp*) [35] zebrafish of either sex were used for this study. We obtained tamalin-mutant mice from NIH [7], and mice of either sex were used for experimentation. The mice were bred in a C57BL/6 background before their use in a specific, pathogen-free facility.

### 4.3. Immunohistochemistry and Wholemount In Situ RNA Hybridization

For the immunohistochemistry analysis, we used the following primary antibodies: rabbit anti-Tamalin (1:200, Novusbio), mouse anti-HuC/D (1:100, Molecular probes), mouse anti-olig2 (1:200, IBL America), and mouse anti-proliferating cell nuclear antigen (PCNA) (1:200, DAKO). To detect fluorescent antibody labeling, we used Alexa 488, 568, 647-conjugated secondary antibodies (1:1000, Molecular Probes). We captured fluorescent pictures of transverse sections using an A1 laser-scanning confocal microscope (Nikon, 1-um z-stack), and wholemount lateral images were obtained using an Eclipse Ti2 Spinning disk confocal microscope (Nikon, 2.5-um z-stack). For in situ RNA hybridization, *tamalin* and the *arf6* open reading frame were cloned into the pGEM T-easy vector (Promega). Not1 and SacII restriction enzyme were used for linearization and transcribed using a DIG labeling combination. Whole-mount or fluorescent in situ RNA hybridization were performed as previously described [36,37]. The primers were designed using the following sequences:

Tamalin forward: 5′-AGGAGTCCTTTGGCTTCG-3′, Tamalin reverse: 5′-GCTTTCCTCCTCCTCCAGAG-3′, Arf6a forward: 5′-ATTTATGCCCAGCCAAC-3′, and Arf6a reverse: 5′-TTCATTGGCGTTAGGATTTG-3′.

### 4.4. TUNEL Assay

We performed terminal deoxynucleotidyl transferase dUTP nick end labeling (TUNEL) assay using a Roche In Situ Cell Death Detection Kit, according to the manufacturer’s instructions. TUNEL staining was performed on 10-um-thick cryosections.

### 4.5. The Generation of CRISPR/Cas9 Mediated KO Zebrafish and Genotyping

We applied the clustered regularly interspaced short palindromic repeats/CRISPR-associated protein 9 (CRISPR/CAS9) system, as described by Wenbiao Chen et al. [38], to produce tamalin KO zebrafish. We used the ChopChop website to create CAS9 target sites for the production of single guide RNA. The target sequence was 5′-CCAAAGTGAGAACTCCGTGGAGA-3′. We purchased the guide RNA and Cas9 protein by TOOLGEN. Embryos at the one cell stage were injected with a guide RNA (500 μg/mL) and Cas9 protein (1000 μg/mL) mixture (F0). Genomic DNA was extracted from 24 hpf embryos (F1), following outcross with F0 and wild type zebrafish to identify a germ line transmitted mutation. Using T7 endonuclease assay, we identified a candidate founder mutant zebrafish. Genomic regions containing the target site were amplified by PCR from the genomic DNA and cloned into the pGEM-T easy vector. We performed DNA sequencing to analyze the regions of mutations. The primers were designed using the following sequences: Tamalin forward: 5′-ACATACGGGCTTCATCACCA-3′, Tamalin reverse: 5′-ACTCGCAGGCCTTAAAGTTG-3′.

### 4.6. Transmission Electron Microscopic Analysis for Zebrafish

Tissues were produced using previously published conventional transmission electron microscopy (TEM) techniques [39]. We anesthetized 10 dpf, 1-, 2-, and 4-month-old zebrafish with tricaine (Sigma) and fixed them for 3 h at room temperature in 4% paraformaldehyde (PFA). 10 dpf larvae and adult spinal cords were preserved in 10% PFA/2.5% glutaraldehyde/0.1 M phosphate buffer and at pH 7.4. Subsequently, the samples were postfixed in 1% osmium tetroxide, dehydrated, and embedded in Eponate-12 resin (Ted Pella). The sections were cut with a Reichert–Jung Ultracut E ultramicrotome (Leica) and stained with toluidine blue before imaging with an Axio microscope (Carl Zeiss). Each resin block yielded a 60-nm-thick slice that was collected on a Formvar-coated slot grid. These slices were stained with uranyl acetate and lead citrate before being photographed with an H-7500 TEM at 80 kV.

### 4.7. Transmission Electron Microscopic Analysis for Mouse

The animals were perfused with 0.9% normal saline before 2% PFA and 2.5% glutaraldehyde in 0.1 M phosphate buffer (pH 7.4). The brains were removed from the skull and stored in the similar fresh fixative overnight at 4 °C. The fixed brains were cut into 1.5 mm-thick coronal sections, including CC or PT and using a coronal brain matrix for mice (Harvard Apparatus). These sections were washed with the 0.1 M phosphate buffer twice, post-fixed in 1% osmium tetroxide for 90 min, dehydrated through an ascending series of ethanol, propylene oxide, and embedded in Epon 812 mixture (Oken Shoji, Tokyo, Japan). Thin sections (70 nm) were prepared using a Leica EM UC6 ultramicrotome (Leica Microsystems, Wetzlar, Germany), mounted on 200-mesh copper grids, stained with 2% uranyl acetate and 1% lead citrate for 5 min each, and observed under a Hitachi H-7650 transmission electron microscope (Hitachi, Tokyo, Japan) at an accelerating voltage of 80 kV.

### 4.8. Behavioral Analysis with Elevated plus Maze (EPM)

The plus maze apparatus was elevated 100 cm above the floor and consisted of two open arms (8 cm × 40 cm), two closed arms (8 cm × 40 cm), and a junction platform (7.5 cm × 7.5 cm). The closed arms were lined with high walls (28.5 cm). At the beginning of the test session, the mice were placed singly in the central area of the maze with their heads oriented toward an open arm. After 10 min of exploration, the mice were returned to their home cages. The number of entries into the open arms and time spent in the open arms were analyzed by tracking the animals using ANY-maze software (Stoelting, Wood Dale, IL, USA).

### 4.9. MPEP Treatments

2-Methyl-6-(phenylethynyl) pyridine (MPEP, tocris) hydrochloride was dissolved in 21.77 mL distilled water to prepare a 1 mM stock solution. The working concentration was 10 μM. Tamalin KO with Arf6-morphant zebrafish were treated with 10 μM MPEP for 2 days [40].

### 4.10. Quantitative Real-Time PCR

Using the Trizol reagent, we extracted total RNAs from the adult spinal cord and FACS-sorted cells. cDNAs were synthesized from the total RNAs using a reverse transcription kit (ImProm-II^TM^ Reverse Transcriptase, Promega). Quantitative RT-PCRs were performed by light cycler. We used 2.5 μL of cDNAs as the template in each reaction combination, along with 0/2 μM forward and reverse primers and 2× FastStart Essential DNA Green Master Mix (Roche). The reactions were performed as follows: 95 °C for 10 min, 95 °C for 10 s, 58 °C for 10 s, and 72 °C for 10 s. We used the following primers: b-actin forward, 5′-AAGGCCAACAGGGAAAAGAT-3′; b-actin reverse, 5′-GTGGTACGACCGGAGGCATAC-3′; Mglur5a forward, 5′-CACACTGAAGGGAATTATGG-3′; Mglur5a reverse, 5′-ACGAGCTTTGGGCAAGTGAC-3′; Mglur5b forward, 5′-ATGGTCATTTTGTGTTCTCTC-3′; and Mglur5b reverse, 5′-GCGTTCATGCACCTTATCTG-3′.

### 4.11. Morpholino Injection and Heat-Inducible Plasmid Construction

To target the start codon of the tamalin, Arf6a, and Arf6b mRNA, we purchased antisense morpholino oligonucleotides (MO) from Gene tools. The MOs were injected into fertilized embryos at the one-cell stage, after being dissolved in nuclease-free water with 1% phenol red. We used the following MOs: Tamalin ATG MO: 5′-TCCGCGTGTCACTCAGTTAGACAGA-3′ and Arf6 ATG MO: 5′-GATCTTGGAAAGCATCTTCCCCATG-3′ [41]. To confirm MO specificity, we produced hsp70:arf6a-mcherry:pA and hsp70:arf6b-mcherry:pA constructs. The ORF of Arf6a and Arf6b with attB1 and attB2 site containing primers were amplified, and the product was cloned into a middle entry vector by a gateway system (Invitrogen). The 5′ entry clone has a heat shock promoter, the middle entry clone has Arf6a, and Arf6b ORF and 3′ entry clone has the mCherry-polyA gene. We used LR clonase II (Invitrogen) for the LR reactions. The primers were designed using the following sequences:

Arf6a attB1 forward: GGGGACAAGTTTGTACAAAAAAGCAGGCTGATTTATGCCCAGCCAACACCATG; Arf6a attB2 reverse: GGGGACCACTTTGTACAAGAAAGCTGGGTAGGATTTGTAGTTGGACGTGAGCC; Arf6b attB1 forward: GGGGACAAGTTTGTACAAAAAAGCAGGCTCATTTATGAACAGTTTACAAGATG; and Arf6b attB2 reverse: GGGGACCACTTTGTACAAGAAAGCTGGGTAAGACTTGTAGTTAGATGTTAAC.

### 4.12. Embryo Dissociation and FACS Sorting

We performed tissue dissociation and FACS sorting with modifications using a Leigh Ann Samsa and Cosacak protocol [42,43]. We anesthetized 3 dpf of *Tg(Tubb:gal4x5uas:egfp)*, Tamalin KO *Tg(Tubb:gal4x5uas:egfp)*, and Arf6 morphant *Tg(Tubb:gal4x5uas:egfp)* in 0.1% tricaine (Sigma) E3 solution in methylene blue. The samples were transferred 50 larvae to 6 tubes per tube (n = 300). Pre-chilled E3 solution was used for rinsing, followed by the addition of 1 mL of deyolk solution (116 mM NaCl + 2.9 mM KCl + 5.0 mM HEPES, set pH at 7.2 + 30 ul of 100 mM phenylmethyl sulfonylfluoride in isopropanol + 1 mL of 10 mM EDTA). All deyolking and dissociation steps were performed on ice to prevent cell degradation. The larvae were dissociated with the dissociation kit (Miltenyi biotec) by incubating for 30 min at 28 °C in a dissociation buffer. We used a 40 μm cell strainer (FALCON) placed in a 50 mL conical tube to filter the cell solution after washing the cell strainer with DPBS/Pen twice. The cell suspension was centrifuged at 1300 rpm for 5 min, followed by supernatant removal. The pellet was resuspended in 1 mL of suspension buffer with bovine serum albumin. We used FACS Melody (BD Biosciences) to sort the dissociated cells for GFP expression.

### 4.13. Adult Spinal Cord Dissection

After 4 months tamalin KO *Tg(Tubb:gal4x5uas:egfp)* zebrafish were anesthetized using 0.1% tricaine in E3 solution. The musculature and skin were removed until the spinal cord was revealed, and it was extracted using fine forceps. The extracted spinal cord was stored in a dissociation buffer.

### 4.14. Statistical Analysis

Graphpad Prism 7 software was used for all statistical analysis. For normally distributed data, we performed the student’s unpaired t-test to compare the two groups. We performed the Mann–Whitney U test for unevenly distributed data. Statistical significance was assessed for all data for the two-tailed probability < 0.05.

## 5. Conclusions

In conclusion, our data suggested that tamalin was associated with Arf6, and it played a crucial role in neuronal and oligodendrocyte survival and myelination through the regulation of mGluR5 in the embryonic and post-embryonic CNS. Abnormal mGluR induces cell death through glutamate toxicity and is involved in various neurodegenerative diseases, including MS, thus necessitating further studies on the role of tamalin and Arf6 in neurodegenerative diseases.

## Data Availability

Data supporting the findings of this study shall be made available in the article and the Supplementary Information Files, or from the corresponding authors upon reasonable request.

## Supplementary Materials

The following supporting information can be downloaded at: https://www.mdpi.com/article/10.3390/ijms232113395/s1.
