# Supplementary material for: Tamalin Function Is Required for the Survival of Neurons and Oligodendrocytes in the CNS
Source: Int J Mol Sci. 2022 Nov 2;23(21):13395. doi: 10.3390/ijms232113395 (PMC9654138; doi:10.3390/ijms232113395)
Supplement: Supplementary file 1 [file ijms-23-13395-s001.zip › ijms-1928881-supplementary.pdf]

Supplementary Figures

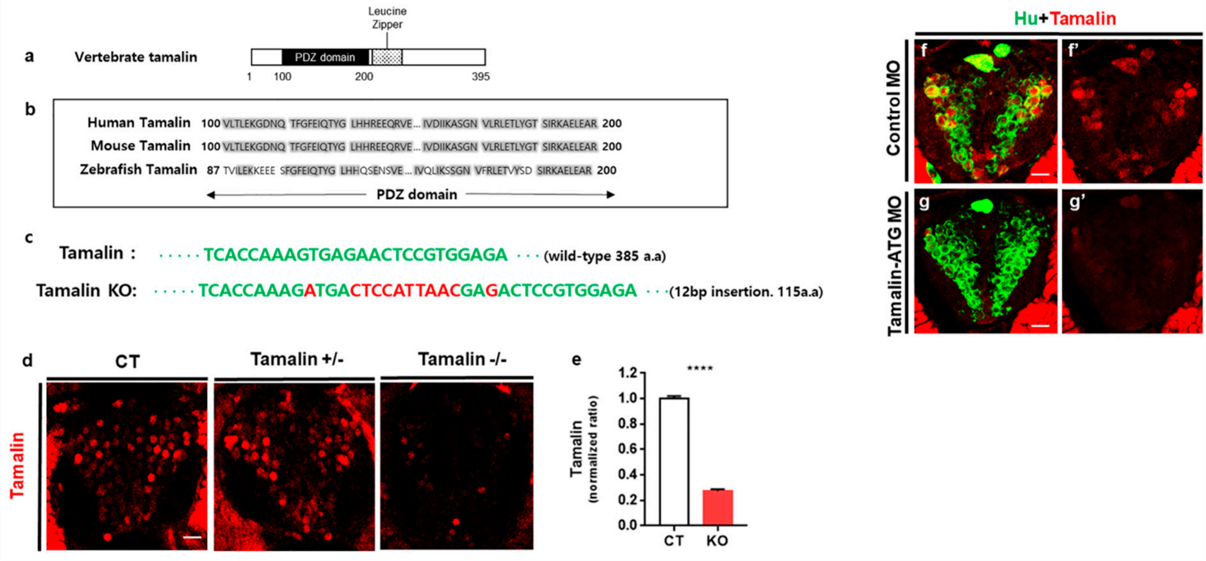

Supplementary Figure S1. The generation of tamalin KO zebrafish and tamalin MO specificity test. (a) Schematic structures of vertebrate tamalin. (b) The amino acid sequence alignment of the PDZ domains of tamalin in different species, displaying identical amino acids in gray boxes. (c) Targeting the PDZ domain with Crispr/Cas9 system causes 12-bp insertion in exon 3, thus generating a premature stop codon and truncated tamalin protein. (d) Immunostaining with anti-tamalin antibody showed that tamalin immunoreactivity almost disappeared in the spinal cord section of tamalin KO zebrafish whereas it was observed in the control and heterozygous tamalin+/- zebrafish. (e) qRT-PCR analysis revealed that tamalin mRNA level was significantly decreased in the tamalin KO zebrafish compared with that in the wildtype. **** $p < 0.0001$. (f-g′) A specificity test of tamalin MO. Transverse sections of the spinal cord of wildtype (f, f′) and tamalin MO-injected embryos (g, g′) labeled with anti-Hu (green) and anti-Tamalin antibodies (red) to detect neurons and tamalin, respectively. The dorsal side is displayed at the top. Scale bars: d-e′, 10 um. KO, knockout; MO, morpholino oligonucleotides; PDZ, post synaptic density protein (PSD95), Drosophila disc large tumor suppressor (Dlg1), and zonula occludens-1 protein (zo-1); and Crispr/Cas9, clustered regularly interspaced short palindromic repeats/CRISPR-associated protein 9.

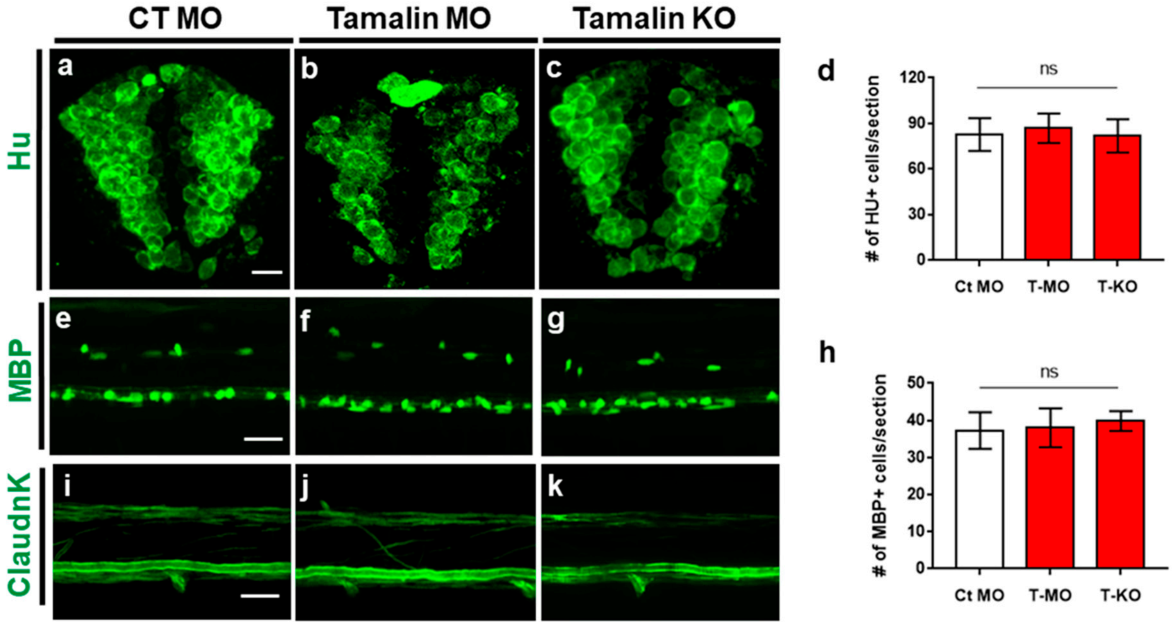

Supplementary Figure S2. Tamalin is not required for neuronal and oligodendrocyte development. (a-c) Transverse sections of the spinal cord of wildtype embryos injected with control MO (CTMO), (a) tamalin MO (b), and tamalin KO embryos (c) labeled with anti-Hu antibody to detect neurons at 3 dpf. The dorsal side is displayed at the top. (d) The quantification of the number of Hu^{+} postmitotic neurons (12 sections from four zebrafish). (e-g) Lateral views of the spinal cord of *Tg(mbp:egfp)* zebrafish injected with CTMO (e), tamalin MO (f), and *Tg(mbp:EGFP)/tamalin*$^{-/-}$ zebrafish at 3 dpf. The anterior side is displayed at the left. (h) The quantification of the *mbp*:$EGFP^{+}$ mature oligodendrocytes (12 embryos). (i-k) Lateral views of the spinal cord of *Tg(ClaudinK:mgfp)* zebrafish injected with CTMO (i), tamalin MO (j), and *Tg(ClaudinK:mgfp)/tamalin*$^{-/-}$ zebrafish (k) at 3 dpf. The anterior side is displayed at the left. Scale bars: a-c, 10um e-k, 20um. ns: not significant.

KO, knockout; MO, morpholino oligonucleotides; dpf, days post fertilization; and EGFP, Enhanced green fluorescent protein.

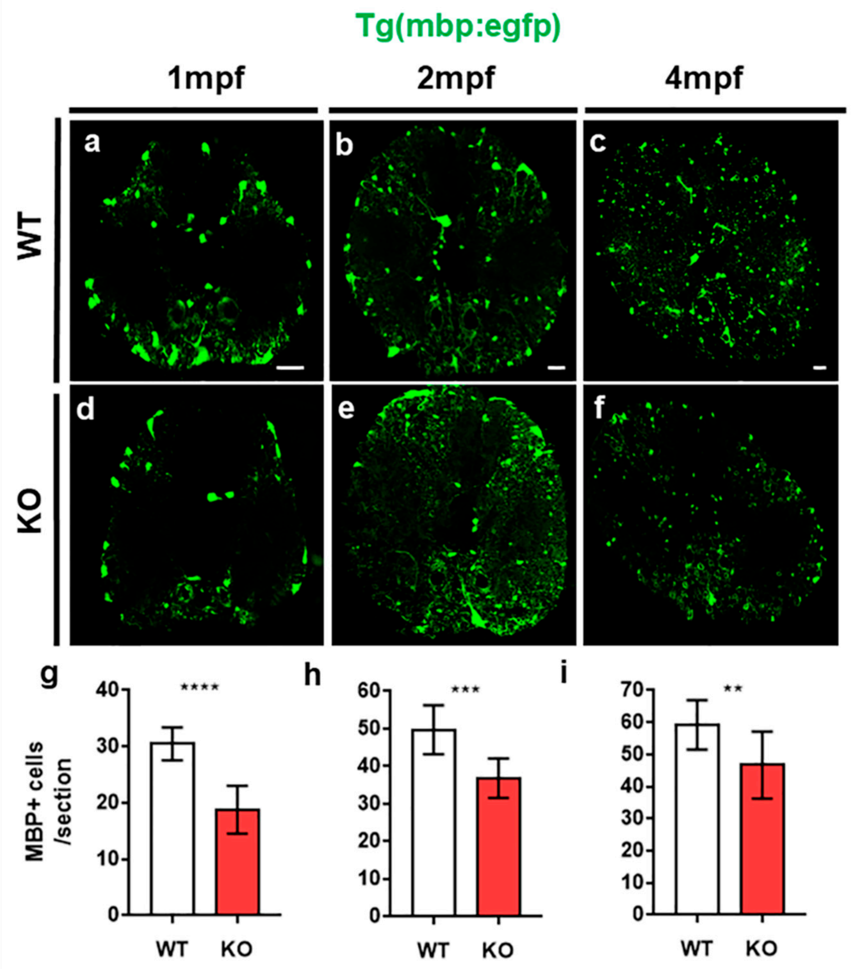

Supplementary Figure S3. The number of mature oligodendrocytes has reduced in the spinal cord of tamalin KO zebrafish at post-embryonic stages. (a-f) Transverse sections of the spinal cord of *Tg(mbp:egfp)* zebrafish (a-c) and *Tg(mbp:EGFP)/tamalin*$^{-/-}$ zebrafish (d-f) at 1 mpf (a, d), 2 mpf (b, e), and 4 mpf (c, f). The dorsal side is displayed at the top. (g-i) Quantification of the number of *mbp*:EGFP^{+} mature oligodendrocytes at 1 mpf (g), 2 mpf (h), and 4 mpf (i) (**p=0.0040 ***p=0.0004 ****p<0.0001, 10 sections from five zebrafish). Scale bars: a-f, 20 um.

KO, knockout; EGFP, Enhanced green fluorescent protein.

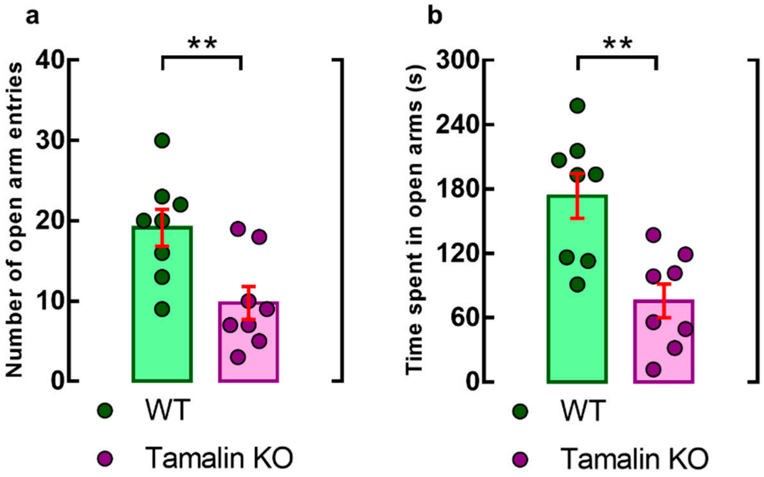

Supplementary Figure S4. Tamalin knockout effects on elevated plus maze test. To examine the role of tamalin in anxiety, mice were tested in an elevated plus maze (EPM). The mice with *tamalin* gene knockout (KO) showed decreased number of open arm entries and time spent in open arms compared with the wild-type (WT) mice in the EPM. (**a)** The number of open arm entries and (**b)** time spent in open arms (s) were measured. $^{**}P < 0.01$. Data are presented as the means ± s.e.m.

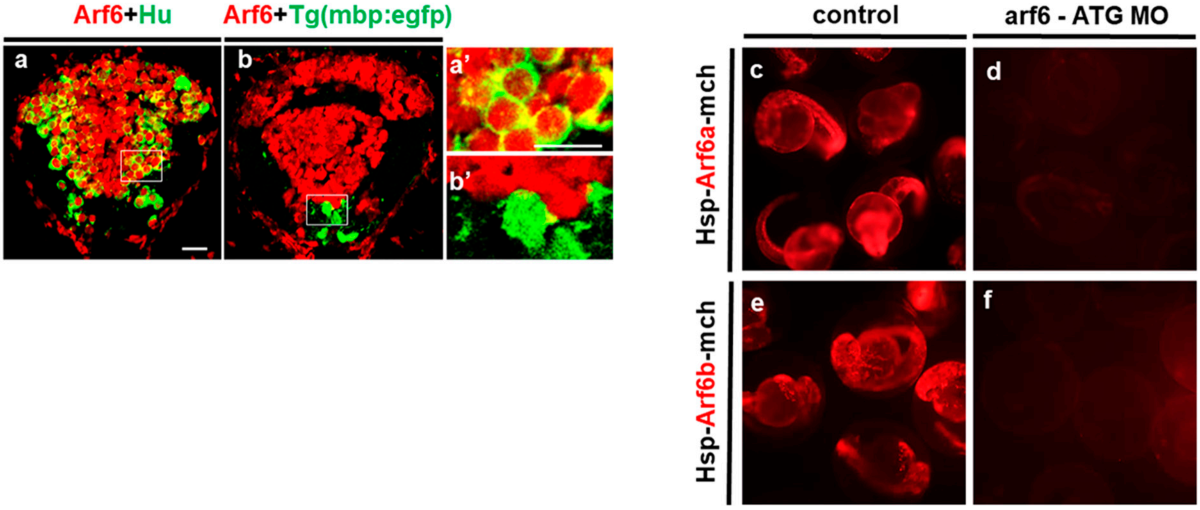

Supplementary Figure S5. Arf6 is expressed in the neurons but not in the oligodendrocytes (a, b). Transverse sections of the spinal cord of the wildtype (a) and *Tg(mbp:egfp)* embryos at 3 dpf. The dorsal side is displayed at the top. Fluorescent *in situ* RNA hybridization with Arf6a mRNA and immunostaining with anti-Hu antibody (a) reveals Arf6 expression in neurons. Contrarily, fluorescent *in situ* RNA hybridization with Arf6 mRNA in the *Tg(mbp:egfp)* embryos reveals that Arf6 is not expressed in the *mbp*:EGFP$^+$ mature oligodendrocytes. (b') High magnification images of the boxes in (a, b). Scale bars: ab', 10 um. (c-f) Specificity test for Arf6 MO. Wholemount fluorescence images of the embryos injected with *hsp:arf6a-mcherry* DNA alone (c) or together with Arf6a MO (d) embryos injected with *hsp:arf6b-mcherry* DNA alone (e) or together with Arf6b MO (f). *Hsp70:arf6a/6b-mcherry* DNAs have been injected into the one-cell-stage zebrafish embryos together with either control MO or Arf6a/6b MO. Injected embryos are heat shocked to induce the expression of exogenous ARF6-mCherry fusion protein. Following heat-shock induction, fluorescence intensity has been measured to determine the level of ARF6a/6b-mCherry fusion protein.

Arf6, ADP-ribosylation factor 6; EGFP, Enhanced green fluorescent protein; dpf, days post fertilization; and MO, morpholino oligonucleotides.
